# SARS-Co-V-2 positive status is associated with a more seriously injured population of trauma patients but not independently associated with worse outcomes of trauma care

**DOI:** 10.5249/jivr.v15i2.1818

**Published:** 2023-07

**Authors:** Bryan G. Maxwell, Andrea Greenlaw, Jeffrey Mako, Megan R. Lundeberg

**Affiliations:** ^*a*^ Departments of Anesthesiology, Legacy Emanuel Medical Center, Portland, OR, USA.; ^*b*^ Department of Trauma Services, Legacy Emanuel Medical Center, Portland, OR, USA.; ^*c*^ Department of Surgery, Legacy Emanuel Medical Center, Portland, OR, USA.

**Keywords:** COVID, Coronavirus, Trauma, Injury

## Abstract

**Background:**

SARS-CoV-2 positive status has been considered a predominantly incidental finding among trauma patients. We sought to examine whether concurrent infection is associated with worse outcomes in a contemporary cohort of injured patients during the COVID-19 pandemic.

**Methods:**

Retrospective cohort analysis of a level I trauma center's institutional registry from May 1, 2020 through June 30, 2021. The prevalence of COVID in the trauma population was compared monthly using prevalence ratios relative to population estimates. Unadjusted cohorts of COVID+ vs COVID- trauma patients were compared. COVID+ patients then were matched on age, mechanism of injury, year, and injury severity score (ISS) with COVID- controls for adjusted analysis with a primary composite outcome of mortality.

**Results:**

Out of n=2,783 trauma activations, n=51 (1.8%) were COVID+. Compared to the general population, the trauma population had prevalence ratios for COVID of 5.3 to 79.7 (median=20.8). Compared to COVID– patients, COVID+ patients had worse outcomes, including a higher proportion who were admitted to the ICU, required intubation, underwent a major operation, and had greater total charges and a longer length of stay. However, these differences appeared related to more severe injury patterns in the COVID+ cohort. In the adjusted analysis, no significant differences between groups in any of the outcome variables were observed.

**Conclusions:**

Worse trauma outcomes in COVID+ patients appear to be correlated to the more substantial patterns of injury observed in this group. Trauma patients have substantially higher rates of SARS-CoV-2 positivity than the local population at large. These results reinforce that this population is vulnerable to multiple threats. They will guide the ongoing delivery of care in shaping the needs for testing, PPE for those delivering care, and the capacity and operational needs of trauma systems that must care for a population with such high rates of SARS-CoV-2 infection.

## Introduction

Treatment of traumatic injury has been one of the few areas of American medicine that has not faced substantial initiatives to delay or postpone care delivery during the COVID-19 pandemic. Trauma systems continue to care for injured patients, and in fact the pandemic has been marked by coincident and concomitant increases in some types of traumatic injury.^1^

Analyses of surgical databases have suggested that SARS-CoV-2 infection is associated with worsened perioperative outcomes even with mild or asymptomatic disease,^2-4^ and consensus guidelines have recommended delaying care when possible.^5^ For surgical care that cannot be delayed, analyses of urgent and emergent procedures demonstrate higher morbidity and mortality.^6^

We sought to use a contemporary cohort at a large level I trauma center to evaluate the hypothesis that SARS-CoV-2 positive status is associated with worsened clinical outcomes in the setting of traumatic injury. We also sought to analyze resource utilization devoted to the care of this population, including the use of imaging modalities, need for surgical intervention, length of stay, and cost of care. 

## Methods 

Legacy Emanuel Medical Center is an urban level I trauma center (one of only two serving the state of Oregon) with 554 beds and approximately 2700 trauma admissions yearly. We queried our institutional trauma registry for all trauma activations from May 1, 2020 (when our system universally began capturing COVID testing) to June 30, 2021 and separated them based on COVID status. COVID status was defined by a positive results of polymerase chain reaction (PCR) testing for SARS-CoV-2 performed at the time of initial presentation to the trauma system. Specific testing assays used in our institution included: standard testing with Roche Cobas 6800 PCR from the beginning of the pandemic through December 22, 2020, at which point we switched to the GenMark ePlex respiratory panel assay; rapid testing with Cepheid GeneXpert 3in1 PCR assays from the beginning of the pandemic through November 2, 2020, when it was converted to the Cepheid GeneXpert 4in1 PCR.

This study received Institutional Review Board approval. We followed all items, as applicable, of the consensus checklist for retrospective cohort studies contained in version 4 of the Strengthening the Reporting of Observational Studies in Epidemiology (STROBE) statement.^7^

SARS-CoV-2 positivity rates in our trauma population were compared to population-level positivity rates using a county-specific CDC public data set [Centers for Disease Control and Prevention, COVID-19 Response. COVID-19 Case Surveillance Public Use Data with Geography (version date: December 20, 2021)] filtered for Multnomah County, Oregon, and our trauma population positivity rate expressed prevalence ratios, i.e. multiples of population risk. For the purposes of this analysis, we assumed a conversion between daily case rates and population-level prevalence of test positivity of 8 days as the median time individuals would test positive (expert opinion by personal communication, David O. Freedman, MD, American Society of Tropical Medicine and Hygiene, Jan 4, 2022). 

Unadjusted analysis was performed with respect to both clinical and outcome variables. The primary outcome was patient mortality. Secondary outcomes included use of computed tomography (CT) imaging, performance of a major operation, length of stay, discharge disposition, and total hospital charges. Use of CT imaging and performance of a major operation were defined using International Classification of Diseases, Tenth Revision, Procedure Coding System (ICD-10-PCS) codes. Discharge disposition was defined as home (including self-discharge against medical advice and discharge to law enforcement), discharge to a skilled nursing or other facility, and death (including hospice).

Adjusted analysis was performed using a matched case-control design. From the cohort of COVID- patients, we created a matched control cohort, using a greedy matching algorithm (“gmatch” SAS macro) for an up to 4:1 match on age (±2 years), year (exact), trauma mechanism (penetrating vs. blunt), and injury severity score (ISS). For matching, ISS was treated as a categorical variable using quartiles based on prior literature demonstrating its nonnormal (positively skewed) distribution and, despite its numeric scale, its unsuitability for treatment as a continuous variable.^8^

Post-hoc qualitative review of COVID+ patients was performed to assess the nature of the COVID infection: recurrent/persistent (evidence of COVID+ testing prior to their trauma admission, suggestive of persistent positivity), incidental (no signs of respiratory compromise apart from those attributable to trauma), or concurrent (respiratory symptoms and/or CT findings present during admission that were possibly attributable to COVID).

Descriptive variables are reported as median [interquartile range (IQR)] and number (percentage) unless otherwise noted. Continuous outcomes were compared using a Mann-Whitney-Wilcoxon test. Binary outcomes were compared using Fisher’s exact tests and odds ratios (OR) with 95 per cent confidence intervals. Analyses were performed using SAS (version 9.4; SAS Institute, Cary, NC, USA) with a predetermined alpha=0.05 for statistical significance.

## Results

Out of n=2,783 trauma activations, we identified a cohort of n=51 (1.8%) patients with COVID [henceforth “COVID+”] and n=2732 (98.2%) without COVID [henceforth “COVID–”].

**Figure 1:**
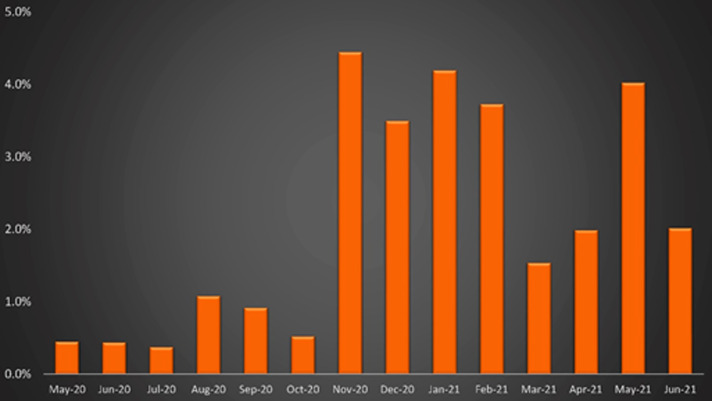
Absolute COVID positivity rates in the trauma population, by month.

**Figure 2:**
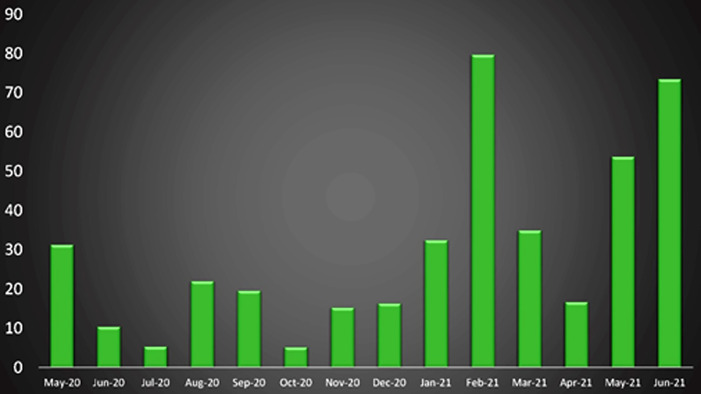
Prevalence ratios of COVID positivity in the trauma population relative to local population estimates, by month.

Patterns of COVID positivity in the trauma population are shown by month in Figure 1 as absolute percentages and in Figure 2 as prevalence ratios compared to estimated positivity rates of the local population at large, which ranged from 5.3 to 79.7 with a median of 20.8.

Demographic and baseline clinical characteristics of the unadjusted cohorts are shown in Table 1.

**Table 1:** Baseline and injury characteristics of the unadjusted cohorts.

	COVID+ (n=51)		COVID– (n=2732)		p
	n	%	n	%	
Age [median (IQR)]	37	(22,54)	46	(25,67)	0.08
Male	36	(70.6%)	1813	(66.4%)	0.65
Race/ethnicity					0.0002
White, non-Hispanic	26	(51.0%)	2046	(74.9%)	
Black	10	(19.6%)	188	(6.9%)	
Hispanic	9	(17.6%)	261	(9.6%)	
Asian/Pacific Islander	0	(0.0%)	68	(2.5%)	
Other/Decline to State	6	(11.8%)	169	(6.2%)	
Positive screen for ethanol	6	(11.8%)	463	(16.9%)	0.20
Positive drug screen	22	(43.1%)	956	(35.0%)	0.53
Trauma Level 1*	23	(45.1%)	590	(21.6%)	0.0002
Mechanism: penetrating	12	(23.5%)	289	(10.6%)	0.009
Injury Severity Score [median (IQR)]	12	(5,19)	9	(5,14)	0.06
Injury Severity Score quartile					0.0027
<5	11	(21.6%)	567	(21.2%)	
5 to 9	6	(11.8%)	829	(13.0%)	
10 to 14	12	(23.5%)	698	(21.6%)	
>14	22	(43.1%)	638	(44.2%)	

* Trauma activations triaged based on pre-hospital criteria as level 1 (more serious) or level 2 (less serious).

Post-hoc qualitative review of the nature of the COVID infection revealed recurrent/persistent prior COVID infection in n=5 (9.8%), incidental COVID infection in n=31 (60.8%), and concurrently symptomatic in n=15 (29.4%). There were no deaths attributed specifically to COVID-related respiratory failure. 

Compared to COVID– patients, COVID+ patients had worse outcomes of the trauma admission (see Table 2). 

**Table 2:** Outcomes of the unadjusted cohorts.

	COVID+ (n=51)		COVID– (n=2732)		p	OR	95% Conf Interval
	n	%	n	%			
CT scan performed	38	(74.5%)	1905	(69.7%)	0.54	1.27	(0.67 , 2.39)
ICU admission	22	(43.1%)	581	(21.3%)	0.0005	2.81	(1.60 , 4.93)
Intubation	14	(27.5%)	344	(12.6%)	0.0046	2.63	(1.41 , 4.91)
Major operation to thorax	13	(25.5%)	251	(9.2%)	0.0007	3.38	(1.78 , 6.43)
Abdominal	9	(17.6%)	133	(4.9%)	0.0009	4.19	(2.00 , 8.78)
Cardiac	1	(2.0%)	25	(0.9%)	0.38	2.17	(0.29 , 16.30)
Thoracic	6	(11.8%)	145	(5.3%)	0.055	2.38	(1.00 , 5.67)
Total charges [median (IQR)]	$68,973	($46,454 , $134,393)	$48,050	($28,673 , $86,278)	0.0005		
Length of stay [median (IQR)]	6	(3, 13)	4	(2, 8)	0.015		
Discharged home*	38	(80.9%)	2116	(81.9%)	0.85	0.94	(0.45 , 1.95)
Mortality	5	(9.8%)	133	(4.9%)	0.11	2.12	(0.83 , 5.43)

* Of patients discharged alive

A higher proportion of COVID+ were admitted to the ICU, required intubation, underwent a major operation, and had greater total charges and a longer length of stay.

Matching successfully resulted in a control cohort of n=231 COVID– patients, with 100% of COVID+ patients successfully matched. Testing confirmed successful balancing between cohorts with respect to age (p=0.84), ISS quartile (p=0.99), and mechanism (p=0.45). Full demographic and baseline clinical characteristics of the adjusted cohorts are shown in Table 3.

**Table 3:** Baseline and injury characteristics of the adjusted cohorts.

	COVID+ (n=51)		COVID– (n=231)		p
	n	%	n	%	
Age [median (IQR)]	37	(22, 54)	37	(22, 56)	0.83
Male	36	(70.6%)	170	(73.6%)	0.73
Race/ethnicity					0.09
White, non-Hispanic	26	(51.0%)	156	(67.5%)	
Black	10	(19.6%)	22	(9.5%)	
Hispanic	9	(17.6%)	29	(12.6%)	
Asian/Pacific Islander	0	(0.0%)	5	(2.2%)	
Other/Decline to State	6	(11.8%)	19	(8.2%)	
Positive screen for ethanol	6	(11.8%)	38	(16.5%)	0.51
Positive drug screen	22	(43.1%)	93	(40.3%)	0.74
Trauma Level 1*	23	(45.1%)	83	(35.9%)	0.26
Mechanism: penetrating	12	(23.5%)	44	(19.0%)	0.45
Injury Severity Score [median (IQR)]	12	(5, 13)	13	(5, 25)	0.58
Injury Severity Score quartile					0.99
<5	11	(21.6%)	49	(21.2%)	
5 to 9	6	(11.8%)	30	(13.0%)	
10 to 14	12	(23.5%)	50	(21.6%)	
>14	22	(43.1%)	102	(44.2%)	

* Trauma activations triaged based on pre-hospital criteria as level 1 (more serious) or level 2 (less serious).

Adjusted analysis of the matched cohorts showed no significant differences between groups in any of the outcome variables of interest (see Table 4 ).

**Table 4:** Outcomes of the matched cohorts.

	COVID+ (n=51)		COVID– (n=2732)		p	OR	95% Conf Interval
	n	%	n	%			
CT scan performed	38	(74.5%)	181	(78.4%)	0.58	1.19	(0.68 , 2.09)
ICU admission	22	(43.1%)	93	(40.3%)	0.75	1.12	(0.61 , 2.08)
Intubation	14	(27.5%)	56	(24.2%)	0.72	1.18	(0.60 , 2.34)
Major operation to thorax	13	(25.5%)	41	(17.7%)	0.24	1.59	(0.78 , 3.24)
Abdominal	9	(17.6%)	25	(10.8%)	0.23	1.76	(0.77 , 4.05)
Cardiac	1	(2.0%)	5	(2.2%)	0.99	1.09	(0.18 , 6.62)
Thoracic	6	(11.8%)	26	(11.3%)	0.99	1.05	(0.41 , 2.70)
Total charges [median (IQR)]	$68,973	($46,454 , $134,393)	$63,581	($34,565 , $125,902)	0.27		
Length of stay [median (IQR)]	6	(3, 13)	5	(3, 10)	0.18		
Discharged home*	38	(80.9%)	170	(82.1%)	0.84	0.92	(0.41 , 2.06)
Mortality	5	(9.8%)	24	(10.4%)	0.99	0.94	(0.34 , 2.59)

* Of patients discharged alive

## Discussion

Our trauma population demonstrated levels of SARS-CoV-2 positivity that were substantially higher than those of the local population: a median of over 20 times higher, with a range of 5- to almost 80-fold. The COVID+ group also had a higher proportion of non-White patients, which is consistent with epidemiologic evidence that SARS-CoV-2 has disproportionately affected communities of color.^9^

We believe this is a striking demonstration of the vulnerability of this population, particularly during a pandemic that has disrupted social services and increased the isolation and fragility of the population most susceptible to injury. Patient-specific vaccination data were not reliably available in this study, but qualitatively, we consistently have observed extremely low levels of vaccination among trauma patients in our institution throughout the vaccine era. 

The substantial virus prevalence also provides strong validation for concerns about workforce exposure that were raised in the pre-vaccine era, during which PPE shortages created strong pressures for staff to care for untested trauma patients without high-level PPE unless and until there was a positive test. Clearly, we see in retrospect than an untested trauma patient had a significantly higher pre-test probability of presenting an exposure risk than the average untested civilian. Daily care of trauma patients created a high cumulative risk of exposure for all providers.

The abrupt increase in the prevalence of COVID in our trauma population observed beginning in November 2020 (see Figure 1) is likely a product of two phenomena. One, this is when case rates in the general population reached their highest levels of the study period. Two (and in large part because of the increased population-level prevalence), this time point coincided with a practice shift in our institution. Prior to December 14, 2020, trauma patients were tested for COVID only if they exhibited symptoms or if an operative intervention was planned (for exposure-risk-stratification and PPE guidance for operating room staff). After this date, every trauma patient was tested on admission.

Post-hoc sensitivity analysis showed no difference in ISSs before and after this date, indicating that the finding that COVID+ patients were more severely injured was not primarily a result of confounding during the selective testing era (e.g. testing to identify positive status only occurred if an injury required an intervention). 

The COVID+ cohort demonstrated patterns of injury that reinforce the observation that this population is vulnerable to multiple threats and risks, as they had a higher ISSs (nearly double the proportion fell into the highest ISS quartile, see Table 1), a significantly greater proportion of penetrating trauma, and a higher likelihood of presenting as the highest triage level (Level 1). COVID+ patients were also younger and more likely to be non-White. 

Outcome differences between the COVID+ and COVID– cohorts (higher proportion admitted to the ICU, higher proportion intubated, higher proportion undergoing a major operation, greater total charges, longer length of stay) all appear to be correlated to the more substantial patterns of injury observed in the COVID+ group, as they did not persist after matching. In adjusted analysis, COVID+ patients did not have a higher likelihood of intubation or ICU admission. Total charges and length of stay were not different, nor was the proportion discharged home.

Focused analysis revealed that most SARS-CoV-2 infection was asymptomatic. The 29% classified as potentially symptomatic concurrent COVID disease is likely an overestimate, since many symptoms (e.g. need for supplementary oxygen) cannot be definitively parsed as from COVID compared to from other factors during the trauma admission. What is perhaps most striking is the absence of any observed phenomena suggesting that COVID+ patient had a significant incidence of subacute respiratory disease that progressed after admission or a greater rate of pulmonary complications from trauma-related care, as mechanical ventilation, ICU admission, and length of stay were no different after adjusting for injury severity and other confounders. Also, we did not observe a longer length of stay or different proportion of patients discharged to home, despite anecdotal concerns that COVID+ status might complicate discharge planning.

These findings exist somewhat in contrast to the suggestion from the surgical literature that even mild or asymptomatic COVID was associated with substantially increased morbidity and mortality at the time of elective or emergent surgery^2,3,6^ though these analyses predominantly reflect the earliest phase of the pandemic and do no fully risk adjust for baseline differences in the cohorts. 

This analysis has a number of limitations inherent to a retrospective, single-institution study. Small cohort size and modest injury severity contributed to limited numbers (e.g. deaths) for analysis. 

There is some imprecision inherent in estimating population prevalence of positive testing, but we used a conservative estimate of 8 days based on conversations with leading infectious disease experts involved in public health leadership during the pandemic, based on observations of serially repeated testing in quarantined travelers. It is likely that this is an overestimate (and therefore the prevalence ratios observed in this study would be even higher) since estimates of median PCR positivity duration were 5 to 8 days in original and alpha variants and have shortened with subsequent variants, including delta.

This study has limited external validity for centers that see more penetrating trauma or different trauma demographics. Matching was able to adjust for the clearest baseline differences, but it is possible some residual confounding was present. The pandemic continues to evolve and features observed in this time period may not translate perfectly to Omicron and subsequent phases.

## Conclusion

SARS-CoV-2 infection was manyfold more common in the trauma population than in the population at large, but it was not independently associated with adverse outcomes. The prevalence of severe COVID appeared very low. These results will guide trauma systems in future planning for the resources needed to care for this vulnerable population – for instance, universal testing, universal respiratory PPE for trauma system providers, and building hospital capacity for cohorting/isolating concomitantly infected patients while providing the necessary care (e.g. intensive care and operating room capacity) for injured patients.
